# A photochemical determination of luminescence efficiency of upconverting nanoparticles

**DOI:** 10.3762/bjoc.15.260

**Published:** 2019-11-11

**Authors:** Baptiste Amouroux, Clément Roux, Jean-Claude Micheau, Fabienne Gauffre, Christophe Coudret

**Affiliations:** 1 Laboratoire des IMRCP, Université de Toulouse, CNRS UMR 5623, Université Toulouse III - Paul Sabatier,118 route de Narbonne, 31062 Toulouse, France; 2 Université de Rennes, CNRS, UMR6226, ISCR, F-35000 Rennes, France

**Keywords:** actinometry, diarylethene, lanthanide, photochemistry, upconverting nanoparticle

## Abstract

Upconverting nanoparticles are a rising class of non-linear luminescent probes burgeoning since the beginning of the 2000’s, especially for their attractiveness in theranostics. However, the precise quantification of the light delivered remains a hot problem in order to estimate their impact on the biological medium. Sophisticated photophysical measurements under near infrared excitation have been developed only by few teams. Here, we present the first attempt towards a simple and cheap photochemical approach consisting of an actinometric characterization of the green emission of NaYF_4_:Yb,Er nanoparticles. Using the recently calibrated actinometer 1,2-bis(2,4-dimethyl-5-phenyl-3-thienyl)-3,3,4,4,5,5-hexafluoro-1-cyclopentene operating in the green region of the visible spectra, we propose a simple photochemical experiment to get an accurate estimation of the efficiency of these green-emitting “nanolamps”. The agreement of the collected data with the previous published results validates this approach.

## Introduction

The photophysical property of converting low-energy light, typically near infrared (NIR), into high energy one thanks to noncoherent photon absorption is called “upconversion”. This phenomenon is exemplified by the lanthanide-based materials [1]. With the rapid developments of nanotechnology, upconverting Ln^3+^-based nanoparticles (UCNPs) have been reported for promising bio-applications [2].

The popularity of this family of photoactive nanocrystals comes from the spectral window that can be used to operate them. Excited at 976 nm or 808 nm, they re-emit over a large range from far-red (802 nm) up to UV in the form of a line spectrum typical of the emissive lanthanides used. The main application foreseen for these nanomaterials is as a substitute of quantum dots [3], since the combination of anti-Stokes emission and noncoherent absorption prevent any luminescence background. Their extreme photostability [4] make them also ideal candidates for single particle tracking. More interestingly, because of the very large range of possible re-emitted energies, UCNPs are now identified as convenient secondary sources of light to trigger locally photoreactions [5–6]. Indeed, the anti-Stokes emission allows bypassing the usual restrictions (power, penetration depth) imposed by the combination of medium composition (organic compound absorbing mostly in the UV–vis range) and the Beer–Lambert law. Moreover, the NIR excitation wavelengths used are much less damaging when biological applications are in sight [7]. “NIR photochemistry”, based on the upconversion phenomenon can find applications in material sciences such as photopolymerization [8], or micellization photocontrol [9], since the excitation wavelength lies in the first transparency window of most biological media, a spectacular range of use in biological sciences has been explored from drug release [10], drug uncaging [11] to photodynamic therapy [12] and optogenetics [13–14]. Inorganic lanthanide based-UCNPs are classically formulated as a mixed fluoride NaREF_4_. Here, RE stands for a cocktail of trivalent rare-earth metal ions containing mostly photophysically inert metals (Y, Gd) and a few percent of “optically active” ions: a sensitizer (often ytterbium) and an emitter (“activator”) such as thulium (UV and blue emissions), holmium (red) or erbium (mostly green). In this solid solution, energy collected by ytterbium at 976 nm is transferred to the less abundant emitting ions. Thanks to lanthanides’ spectroscopic properties (regular level spacing and long excited states lifetimes), one emitting ion can undergo several energy transfer processes before relaxing radiatively [15], making the overall process fundamentally different form second harmonic generation or two-photon absorption. Furthermore, it has the following consequences: (i) the intensity of each line is power-dependent upon the excitation laser power, this latter point being made clear upon plotting each line intensities vs laser power in a log–log plot, (ii) the intensities of the upconversion emission lines are less and less intense as the emitted energy increases, (iii) the intensities of the emission lines but not their wavelength vary with the UCNP size, as the surface quenching becomes the most efficient deactivation path for small nanoparticles. Therefore, the assessment of the upconversion quantum yields (UCQY) is a hot topic as these depend on the size, the excitation power and the formulation of the nanocrystal.

This issue is classically addressed using physical measurements, therefore requiring complex equipment. Most of these assessments are achieved via the use of integration spheres [16–19]. The challenges are to cope with a large spectral range, the variable excitation power and, because UCQY are usually very small, to handle a large energy contrast between incident beam and collected emission. Fully built equipment to carry out UCQY determination are only starting to be developed commercially (Jasco, Hamamatsu). A more sophisticated approach involves microscopic techniques, enabling one to determine UCQY even at the single NP scale. A seminal report was published in 2013 by Nadort et al. [20] describing the measurement of the luminescence of Er-doped UCNPs at the single NP or cluster level after identification by TEM. Yet, this type of work has remained isolated. Moreover, in these conditions, the nanoparticles do not work in conditions close to their foreseen applications. As we became interested in the design of such nanoparticles [21], we envisioned a “chemical approach” of this measurement problem.

**Figure 1:**
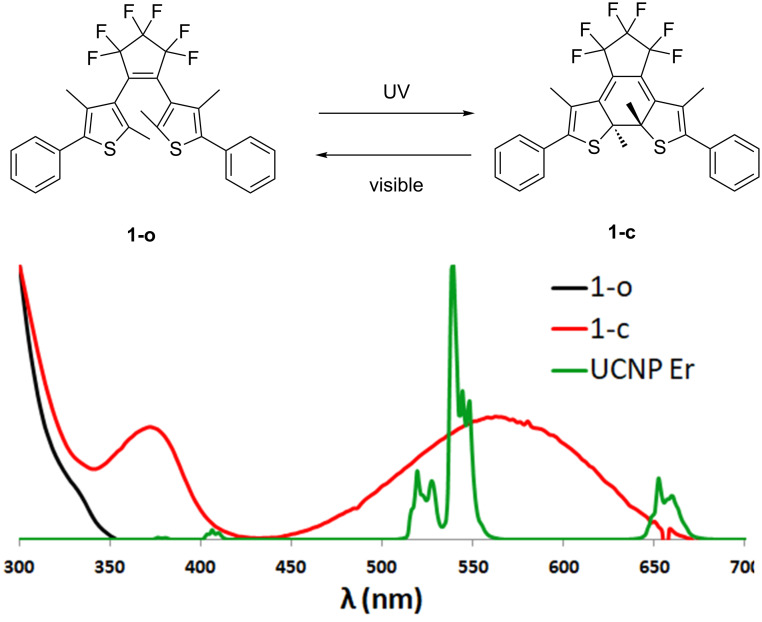
Top: photoisomers of diarylethene **1**, bottom: spectral overlaps between the **1-o** (black line), **1-c** (red line) UV–vis absorption spectra and the Er-UCNP emission spectrum (green line).

The chemical measurement of light intensity is called actinometry and relies on the exposure of a fully standardized photosensitive compound to the light to be measured [22]. The rate of the photochemical transformation is then used to retrieve the light intensity of the beam exciting the solution. Compared to physical radiometry, actinometry is directly transposable to the monitoring of photochemical transformations as it originates from the very same concept and can be performed in the same experimental conditions. It is also adapted to turbid mixtures and can be extended to polychromatic sources. Since the recent renewal of photochemistry caused by the use of LEDs and microfluidic devices, actinometry has become a convenient tool to parameterize the performances of photoreactors [23–25]. Actinometer choice is guided by the operating conditions and by the spectral overlap between the compound and the source. The emission of erbium-containing UCNPs (Er-UCNPs) is dominated by a pair of green (520 nm and 540 nm) and red band (655 nm). In this part of the electromagnetic spectrum, very few actinometers are available. Beside inorganic compounds such as Reinecke salt (ammonium diamminetetrakis(thiocyanato)chromate(III)), photochromic dyes have been proposed for such a purpose, mainly from the azobenzene, fulgide or diarylethene families [22]. The latter two are particularly attractive for visible light wavelengths above 400 nm. However, their use is conditioned by their availability and reliability. Recently, an accurate determination of photochemical quantum yields (QY) [26] was achieved for a commercially available diarylethene 1,2-bis(2,4-dimethyl-5-phenylthien-3-yl)-3,3,4,4,5,5-hexafluoro-1-cyclopentene, labelled **1**. Since then, this dye has been used as actinometer in the visible range (Figure 1) [25,27].

Switching of such diarylethene dyes in both directions (ring closure/coloration or ring opening/discoloration) by UCNPs has been documented for years, with a seminal work reported in 2009 by the team of Branda [28]. In the following we will show how this photochromic compound can be used to give a reasonable quantitative estimation of the upconversion phenomenon. In particular, we will exploit the ring-opening reaction since only the closed form **1-c** presents a good spectral overlap with the visible emissions of the Er-UCNPs.

In order to achieve a “user friendly” quantitative measurement of the light emitted by the nanoparticles, we have chosen to mix together the nanoparticles and the actinometer.

## Results and Discussion

### Upconverting nanoparticles

Hydrophobic nanoparticles were prepared by adapting the standard reported procedure of Li and Zhang (details in Supporting Information File 1) [29]. Briefly, key points are: (i) the in situ preparation of metal oleate from their corresponding chloride, (ii) the introduction of the sodium and fluoride ions as two methanol solutions of respectively NaOH and NH_4_F via separate syringe pumps (according to Zhai et al. [30]) and, after volatile solvents removal, (iii) the high temperature crystallization step for 90 minutes. Spherical nanoparticles of 21.8 ± 1.3 nm were collected. Crystal quality was assayed by XRD and only the hexagonal β-phase could be detected (Supporting Information File 1). These particles are kept well dispersed in cyclohexane.

### Photolysis experiments

**Figure 2:**
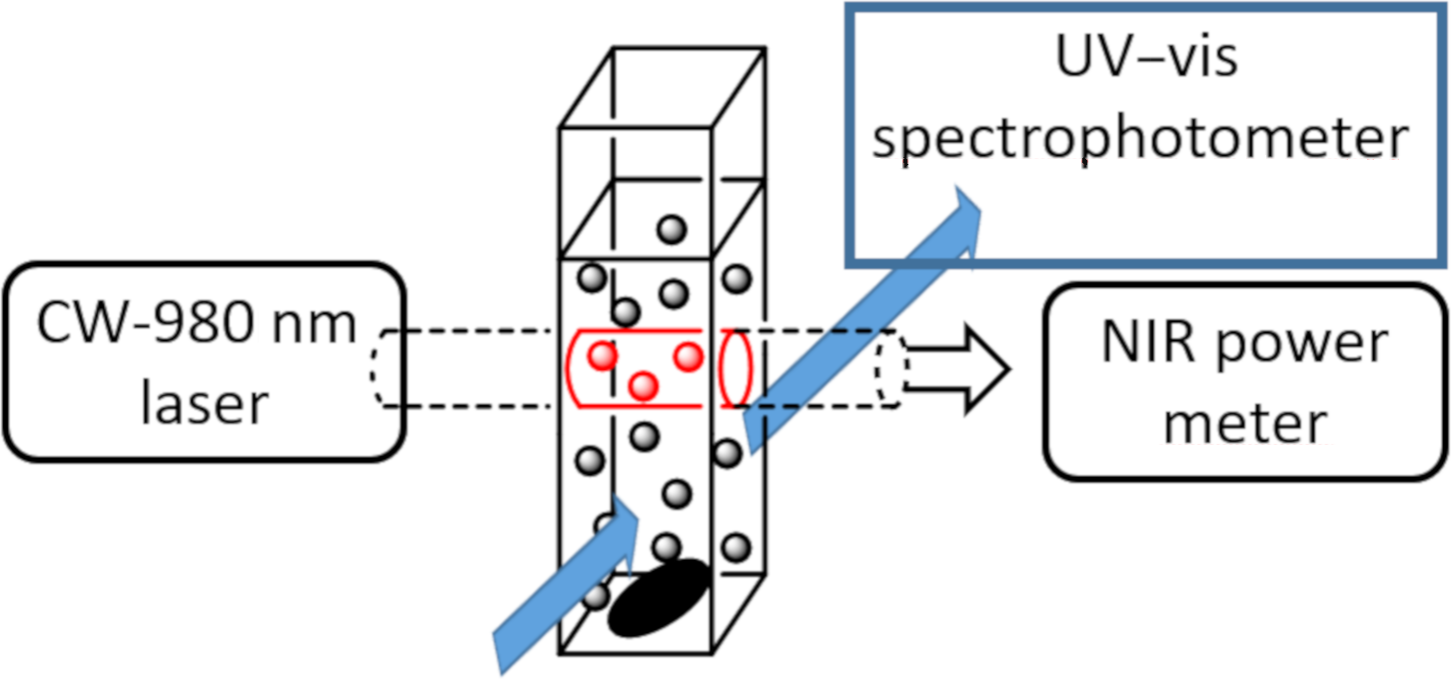
UCNPs (black dots) are irradiated inside the cylindrical CW 976 nm laser beam. Absorbed laser power is recorded with a power-meter. The UV–vis spectrophotometer axis is perpendicular to the laser beam.

The description of the setup is summarized in Figure 2. The sample in a thermostated quartz cuvette was irradiated with a fibered, collimated CW 976 nm-laser beam. The transmitted laser intensity was measured using a calibrated power-meter. This measurement informed us about the fraction of light effectively absorbed by the medium and also the possibility of particle sedimentation. All this set-up was placed inside a UV–visible spectrophotometer (Figure 2).

Using cyclohexane as a common solvent for both diarylethene **1** and UCNPs, we have chosen to work on mixtures of the freshly prepared actinometer **1-c** and nanoparticles. Practically, the preparation of the **1-o/c** solution was achieved using bench-top UV source (TLC lamp), either on the UCNP-**1** mixture or before mixing the dye with the UCNPs. Concentrations were standardized prior the photolysis experiments using published data (ε(**1-c**) 562 nm = 10900 L mol^−1^ cm^−1^ [26] and ε(**Yb**)976nm = 3.1 L mol^−1^ cm^−1^). All the parameters used are gathered in Table 1. Actinometer absorbance changes were continuously monitored by the spectrophotometer [31]. Care has been taken to assess that the cuvette was sufficiently stirred [32], and that the actinometer was neither sensitive to the spectrophotometer measuring beam (laser off) nor to the NIR laser beam in the absence of UCNPs (see Supporting Information File 1).

**Table 1:** Parameters of UCNP used in the photolysis experiment.

parameter	symbol	unit	value

**1-c** concentration	[**1-c**]	mol L^−1^	2.12 × 10^−4^
UCNP concentration	[UCNP]	NP L^−1^	1.18 × 10^16^
volume of the solution	V	L	1.96 × 10^−3^
**1-c** Absorbance at 540 nm (irradiation)	Abs_540_	–	2.07
absorbance of UCNP solution at 976 nm	Abs_976_	–	0.0014
laser power at 976 nm (NIR)	*P*	W	4.7
laser beam section		cm^2^	9.6 × 10^−2^
laser power density at 976 nm (NIR)		W cm^−2^	49

**Figure 3:**
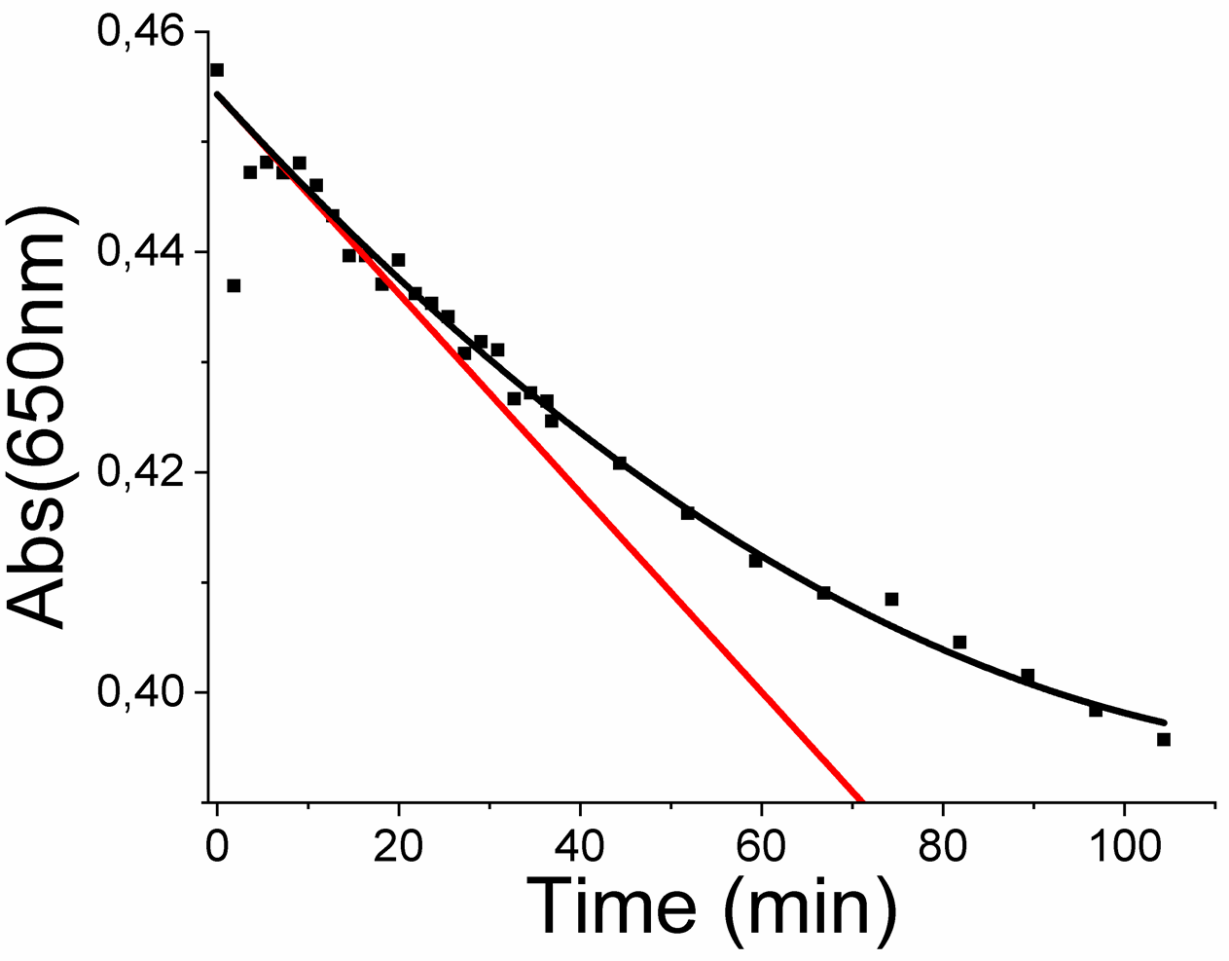
Kinetic trace at 650 nm under CW 976 nm laser at 4.71 W. Initial slope (red line) was determined on the 2nd order polynomial fit of experimental points (dark curve).

Upon 976 nm irradiation, a clear-cut decrease of the absorbance in the visible range can be monitored. Typical kinetic traces were recorded at 650 nm and the data was processed in order to obtain the initial rate of the photoreaction (Figure 3).

Beside these experiments, controls were made to rule out the possibility of thermal effect (irradiation of the actinometer alone with the 976 nm laser) or the possible effects of the spectrometer light source (no laser applied). Lower laser powers were not attempted in order to keep sufficient sensitivity and/or a reasonable reaction time. Data are gathered in Table 2 (vide infra) and in Supporting Information File 1.

### Data treatment

#### The upconversion light source

Unlike two-photon excitation that requires very high local power density, the upconversion process is based on multiple, noncoherent, “single photon” successive absorptions. As the molar extinction coefficient of the sensitizer ytterbium is weak (ca. 3 mol L^−1^ cm^−1^), the exciting beam is moderately attenuated as it crosses the colloidal suspension. Therefore, UCNPs are excited over the entire portion of the 976 nm laser beam that crosses the sample: the resulting visible light source can be considered as a cylinder having for base, the laser section, and for length, the laser path through the cuvette (Figure 2). To compute the number of “active“ nanoparticles, we measured the absorbance A_976_ of the colloidal suspension at 976 nm by measuring the laser intensity that crosses the sample holder, with and without the NP’s suspension. This absorbance is solely due to the ytterbium ions, therefore one can compute the number of Yb atoms *n*_Yb_ inside the beam volume *v* as:

[1] $$n_{\text{Yb}}=\frac{v}{\varepsilon_{\text{Yb}}l}N_{\text{A}}{\text{Abs}}_{976}$$

where ε_Yb_ is the ytterbium atomic molar extinction coefficient at 976 nm (3.1 L mol^−1^ cm^−1^), *l* the optical path crossed by the laser beam (1 cm), *N_A_* is Avogadro’s number. The number of nanoparticles inside the laser beam *n*_NP_ can be determined knowing *N*_Yb_ the number of ytterbium per nanoparticle:


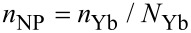


*N*_Yb_ and *N*_Er_ (*N*_Er_: number of erbium atom per particles) can be derived from the number of RE atoms per NP, itself computed from TEM and XRD measurements taking into account the nanoparticles size (volume ≈ 5400 ± 1000 nm^3^), unit cell volume (107.6 Å^3^) and number of NaREF_4_ per unit cell (*Z* = 1.5).

#### The DAE photobleaching experiments

From the spectral overlap one can notice that only the 540 nm erbium line will be the useful one: the UCNP-emission can be considered as quasi-monochromatic within the closed DAE (**1-c**) spectral range. At this wavelength, the value of the ring-opening QY Φ_co_ of actinometer **1** is taken as 0.02, using the calibration curve by Sumi et al. [26].

Monochromatic actinometry is typically ran in a continuously stirred reactor and relies on the following equation:

[2] $$-\frac{\text{d[}1\text{-}c\text{]}}{\text{d}t}=\Phi_{\text{co}}I_{\text{a}}$$

where d[**1-c**]/dt is the rate of consumption of the DAE closed form in mol L^−1^ s^−1^, Φ_co_ is the ring opening quantum yield, i.e., the number of events divided by the number of photons absorbed and *I*_a_ is the rate of photon absorption, in mol *L*^−1 ^*s*^−1^, i.e., the photon flux per volume of solution to be measured. Note that for a given reactor of volume *V*, the photon flux per volume of solution is related to the photon flux *J* by a simple multiplication *J* = I × V. The difficulty is then to relate the rate of absorbed photons *I*_a_ to the incident photon flux *J*_0_ emitted by the UCNPs. A way to circumvent this issue is to adapt actinometer solution absorbance to the reactor used. Indeed, a light-absorbing solution is characterized by its “optical thickness” [23] *L* defined from the rewritten Beer–Lambert law (Equation 3)

[3] $$I=I_{0}10^{-\frac{I}{L}}$$

as

[4] $$L=\frac{\text{1}}{{\text{ε}}_{\text{540}}\text{[}1\text{-}c\text{]}}$$

*L* is therefore the inverse of the absorbance measured for an optical path of 1 cm (Equation 4). For *l* = *L*, *I* = 0.01 × *I*_0_: more than 99% of light is thus absorbed. In our case, we have chosen to use a sufficiently concentrated **1-c** solution so that all the emitted photons are supposedly absorbed. Indeed, an absorbance at 540 nm of 2.07 (over 1 cm) gives a characteristic length of 0.48 cm, comparable to the dimensions of the cuvette: practically no green light escapes the photoreactor.

Under these conditions the actinometric equation becomes

[5] $$-\frac{\text{d[}1\text{-}c\text{]}}{\text{d}t}\approx \Phi_{\text{co}}I_{\text{0}}$$

so the flux in photon per second emitted by the source is:

[6] $$J_{0}=-\frac{N_{\text{A}}}{\Phi_{\text{co}}}v\frac{{\text{dAbs}}_{650}}{{\text{ε}}_{\text{650}}\text{d}t}$$

where *V* is the total volume of the DAE solution and the monitoring optical path is 1 cm. Finally, the average upconversion-QY, Φ_UC_ can be estimated by the ratio

[7] $$\Phi_{\text{UC}}=\frac{J_{0}}{J_{\text{a}}^{\text{NIR}}}$$

where *J*_0_ is the above measured photon flux and *J*_a_^NIR^ is 976 nm laser photon flux absorbed by the nanoparticles:

[8] $$J_{\text{a}}^{\text{NIR}}=J_{0}^{\text{NIR}}(1-10^{-{\text{Abs}}_{976}})=P\frac{\lambda_{976}}{hc}(1-10^{-{\text{Abs}}_{976}})$$

where *P* is the laser power in Watts and *J*_0_^NIR^ the NIR photon flux. Additionally, one can access the number of emitted photons per particles *J*_0_/*n*_NP_ (in photon s^−1^), or, using the energy of a 540 nm photon, to the emitting power of a single nanoparticle

[9] $${℘}^{\text{NP}}=\frac{J_{0}hc}{n_{\text{NP}}\lambda_{540}}\text{ (in Watt)}$$

and the number of emitted photons per erbium atom *J*_0_/*n*_Er_ in photon s^−1^.

All of these numbers are gathered in Table 2, more detailed calculations are provided in Supporting Information File 1.

**Table 2:** Obtained results.

parameter	symbol	unit	value

**1-c** bleaching rate	-d[**1-c**]/d*t*	mol L^−1^ s^−1^	6.78 × 10^-9^
**1-c consumption**		**molecule s^−1^**	**8.00 × 10^12^**
upconversion photon flux at 540nm	*J*_0_	photon s^−1^	4.00 × 10^14^
incident NIR photon flux	*J*_0_^NIR^	photon s^−1^	2.31 × 10^19^
absorbed NIR photon flux	*J*_a_^NIR^	photon s^−1^	7.45 × 10^16^
**up-conversion QY**	**Φ_UC_**	**–**	**0.54%**
number of NPs inside the laser beam	*n*_Yb_	NP	1.14 × 10^12^
number of emitted photons per erbium atom	*J*_0_/*N*_Er_	photon s^−1^	0.24
**number of emitted photons per NP’s**	***J*_0_/n_NP_**	**photon s^−1^**	**350**
**power per NP’s**	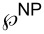	**W**	**1.29 × 10^−16^**

The as-determined quantum yield is in good agreement with measurements obtained on bulk samples by using integrating spheres [33], and the order of magnitude of the emissive power of a single NP is close to what was achieved by microscopy on nanoparticles of similar composition but at a higher laser power (49 × 10^−16^ W under 976 nm irradiation at 260 W cm^−2^) and with a larger size (70 nm instead of 21.8) [20]. One can be surprised by the rather low number of photon emitted per second and per NP: one erbium center emits in average one photon every four seconds. This can be understood as lanthanides’ excited states are long lived and also because the production of one green photon requires three energy transfer steps from excited ytterbium ions. Despite this very weak emission rate, such nanoparticles can be used to induce local photochemistry. Thus, the group of Zvyagin has developed an in situ photodynamic therapy using quite large particles (70 nm) [34] and recruiting the flavin-containing coenzymes as ^1^O_2_ sensitizers. In the skin, typical number of dyes per femtoliter is expected to be 750. This would correspond to an absorbance of 0.0014 in 1 cm of pure water according to a molar extinction coefficient of ca. 11300 L mol^−1^ cm^−1^. To mimic such a situation, we have designed an experiment with larger nanoparticles (35 nm) and dilute **1-c** dye: an absorbance at 540 nm of 0.11 ([**1-c**] = 1.14 × 10^5^ mol L^−1^) corresponds to number of dyes of 6800 molecules per femtoliter. The photoswitching of the actinometer **1-c** was clearly observed (Supporting Information File 1) and an initial “bleaching activity” of 20 dyes per NP and per second could be calculated by dividing the bleaching rate by the number of particles within the laser beam. However, is it very difficult to derive the emitted flux *J*_0_ for this reactor geometry: with a characteristic length *L* of 9 cm, most of the light escapes the cuvette and no simplification can be done. Thus, privileging the spectral information (clear UV–vis spectra are indeed monitored) lead to a loss of information; another photoreactor design would then be necessary.

## Conclusion

We have demonstrated that the chemical approach of a light flux measurement could also be employed for assessing the efficiency of unusual light sources as small as the nanolamps that are upconverting nanoparticles. The observed results are in agreement with published data which is remarkable as the here-described methodology can been run with limited lab equipment. The technique is robust and simple to operate. Concerning the use of single-UCNP as nanolight sources, the emitted flow of photon is rather sparse but yet relevant biological signals could be triggered. This study shows the interest to use P-photochromic dyes as actinometer. Extension to blue emitting UCNPs would however require a suitable dye for the 400–500 nm spectral window, to be found probably in the “inverse DTE” family [35] or in the photodissociable family [36].

## Supporting Information

File 1 Experimental details about the UCNPs syntheses, characterizations, photolysis experiments and detailed calculations.
