# Supplementary material for: A photochemical determination of luminescence efficiency of upconverting nanoparticles
Source: Beilstein J Org Chem. 2019 Nov 11;15:2671–7. doi: 10.3762/bjoc.15.260 (PMC6880820; doi:10.3762/bjoc.15.260)
Supplement: File 1 — Experimental details about the UCNPs syntheses, characterizations, photolysis experiments and detailed calculations. [file Beilstein_J_Org_Chem-15-2671-s001.pdf]

BEILSTEIN JOURNAL OF ORGANIC CHEMISTRY

Supporting Information

for

A photochemical determination of luminescence efficiency of upconverting nanoparticles

Baptiste Amouroux, Clément Roux, Jean-Claude Micheau, Fabienne Gauffre and Christophe Coudret

Beilstein J. Org. Chem. **2019,** *15,* 2671–2677. doi:10.3762/bjoc.15.260

Experimental details about the UCNPs syntheses, characterizations, photolysis experiments and detailed calculations

License and Terms: This is a supporting information file under the terms of the Creative Commons Attribution License (http://creativecommons.org/licenses/by/4.0). Please note that the reuse, redistribution and reproduction in particular requires that the authors and source are credited.
The license is subject to the *Beilstein Journal of Organic Chemistry* terms and conditions: (https://www.beilstein-journals.org/bjoc)

Table of contents

1- General Procedure for the preparation of 21.8 nm- UCNP batch S2

2- UCNP's stoichiometry datas. S2

3- TEM analysis of UCNP batch. S3

4- XRD analysis of UCNP batch. S4

5- Photochromic actinometer S4

6- Discoloration of DAE induced by 21.8 nm-UCNP S5

7- General procedure for the preparation of 35 nm- UCNPs S6

8- Discoloration of DAE induced by 35 nm-UCNP. S6

9- Control experiments. S9

10- Summary table S9

11- References S10

1- General Procedure for the preparation of 21.8nm-UCNP batch

UCNP were prepared adapting the protocols of Li and Zhang[2] and Zhai et al.[3] Rare earth (RE) chloride hydrates were mixed in a oleic acid (OA): octadecene (ODE) (7:38) mixture and heated to 160 °C under Ar flush until a clear solution was obtained. To the cooled reaction mixture were slowly added freshly prepared solutions of NaOH (0.326 mM) and NH_4F (0.5 mM) in MeOH via syringe pump at 0.4 mL min^{-1}. Volatile compounds were then removed under Ar flush then the reaction mixture was brought to 300–310 °C for 90 minutes. Timer was started when temperature reached 300 °C. It should be noted that temperature never reached 310 °C but stayed at around 305 °C.

2- UCNP's stoichiometry datas

	UCNPs
Matrix	*$NaYF_4$*
n(RE)	*11.6 mmol*
n(Y)	*7.9 mmol (68% RE)*
n(Yb)	*3.5 mmol (30% RE)*
n(Yb)	*0.23 mmol (2% RE)*
n(Na)/n(RE)	*4*
n(F)/n(RE)	*5.5*
V(OA) mL	*35*
V(ODE) mL	*190*
{Na+F} addition	*Separated addition via double syringe pump*
Diameter	*21.8 nm*
Area	*$1.49 * 10^3$ nm²*
Volume	*$5.42 * 10^3$ nm^3*
Unit cell volume U($NaYF_4$:Yb,Er)	*107.6 $Å^3$*
Z	*1.5*
N(Yb/NP)	*22670*
N(Er/NP)	*1510*

3- TEM analysis of UCNP batch

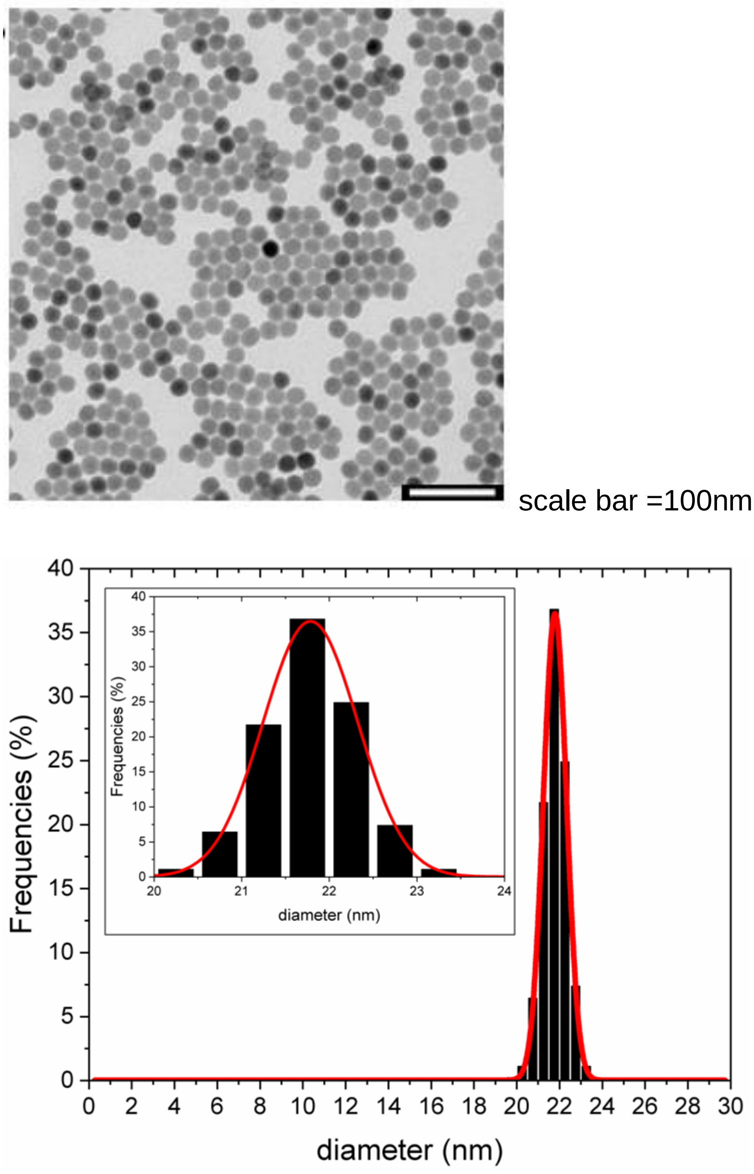

Figure S3 : TEM analysis and size distribution of the studied UCNPs

UCNP synthesized are homogeneous in size and shape. We have assimilated them to a pseudo-sphere of a diameter of 21.8 ± 1.3 nm (average size ± FWHM)

4- XRD analysis of UCNP batch

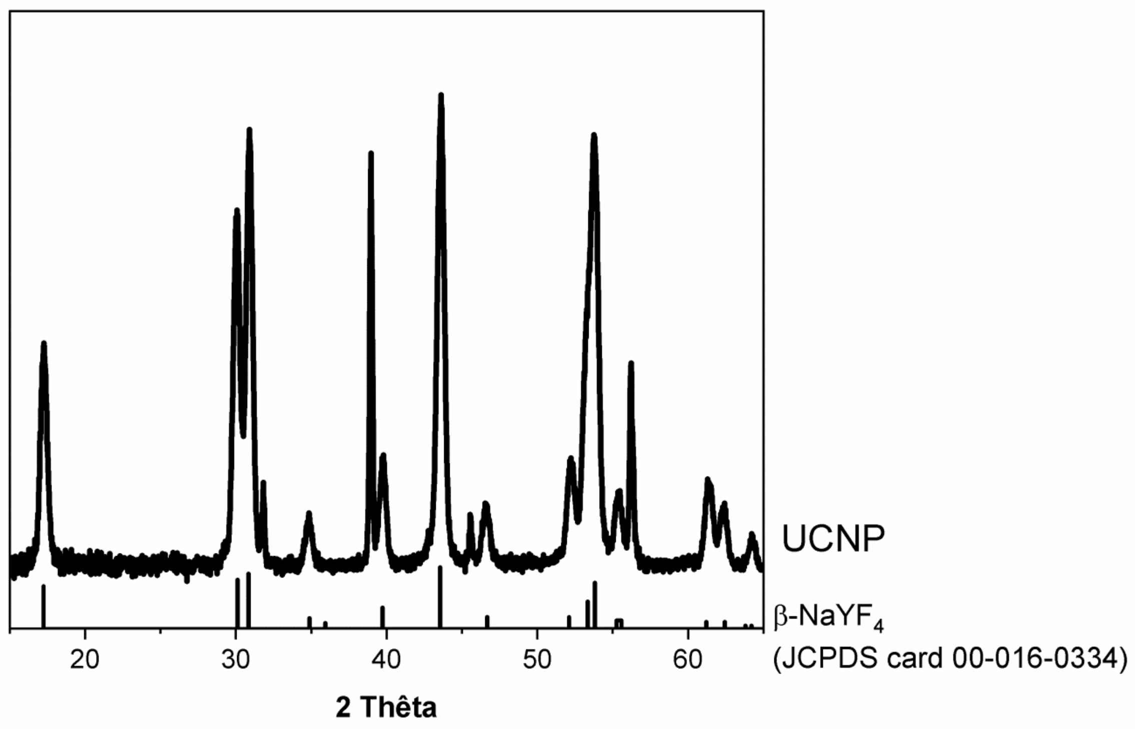

Figure S4 : XRD analysis of the studied UCNP and comparison with the standard hexagonal phase β-$NaYF_4$ (JCPDS card 00-016-0334) .

5- Photochromic actinometer

1,2-Bis(2,4-dimethyl-5-phenyl-3-thienyl)3,3,4,4,5,5-hexafluoro-1-cyclopentene (≥98%, CAS: 172612-67-8) was obtained from TCI chemicals and used as received. The concentration of **1-c** was determined using the published value of its molar extinction coefficient at 562 nm (10900 L $mol^{-1}cm^{-1}$). [1]

6- Discoloration of DAE induced by 21.8 nm-UCNP

Quartz cell containing the mixture of DAE and UCNP (21.8nm) was placed in UV–visible spectrophotometer. Absorbance spectra were recorded in a kinetic mode, while the cell was irradiated from the side by a fibered collimated NIR laser.

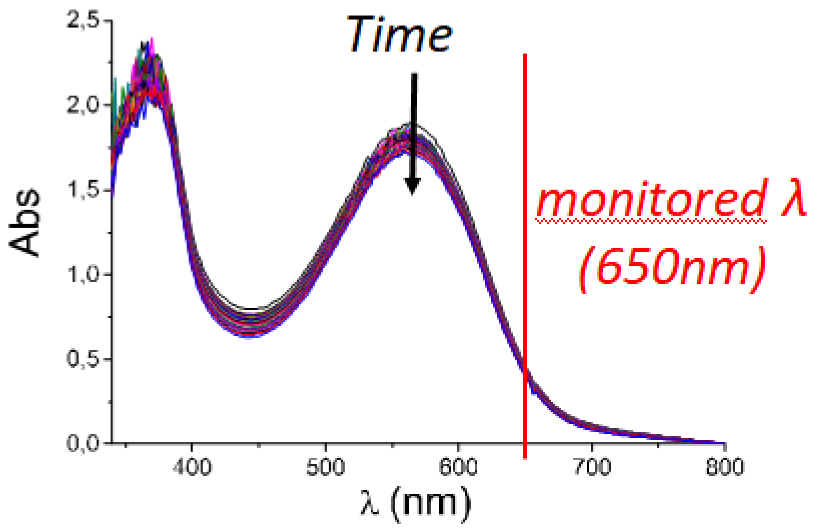

Figure S6 : DAE discoloration induced by UCNP under 976m CW laser at 4.1 W (49 W cm^{-2}).

This high absorbance has been selected to ensure total light absorption at 540 nm. A convenient parameter is the characteristic "optical thickness"[4] L defined from the rewritten Beer-Lambert law:

$$I = I_0 10^{-\frac{l}{L}}$$

as

$$L = \frac{1}{\varepsilon_{540}[1c]}$$

L is therefore the inverse of the absorbance measured for an optical path of 1cm. Thus, an absorbance at 530nm of 2.1 (over 1cm) gives a characteristic length of 0.47 cm, comparable to the dimensions of the cuvette. On the other hand, a shift of the observation wavelength was necessary to optimize the monitoring conditions.

7- General procedure for the preparation of 35nm -UCNPs.

Larger $NaYF_4$:Yb,Er UCNPs were prepared by applying the protocol described by Li and Zhang[3], using a doping molar ratio of 80% Y, 18% Yb, 2% Er. TEM analyses give an average size of 35 nm

8- Discoloration of DAE induced by 35nm-UCNP

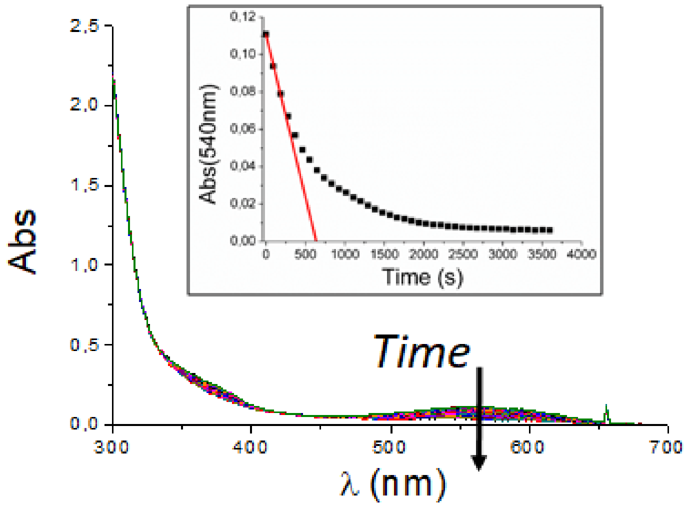

Figure S8-1: Photoisomerization of DAE induced by UCNP emission was monitored in diluted conditions: Abs(500–600nm) ≈ 0.15. Complete kinetic is represented in the insert.

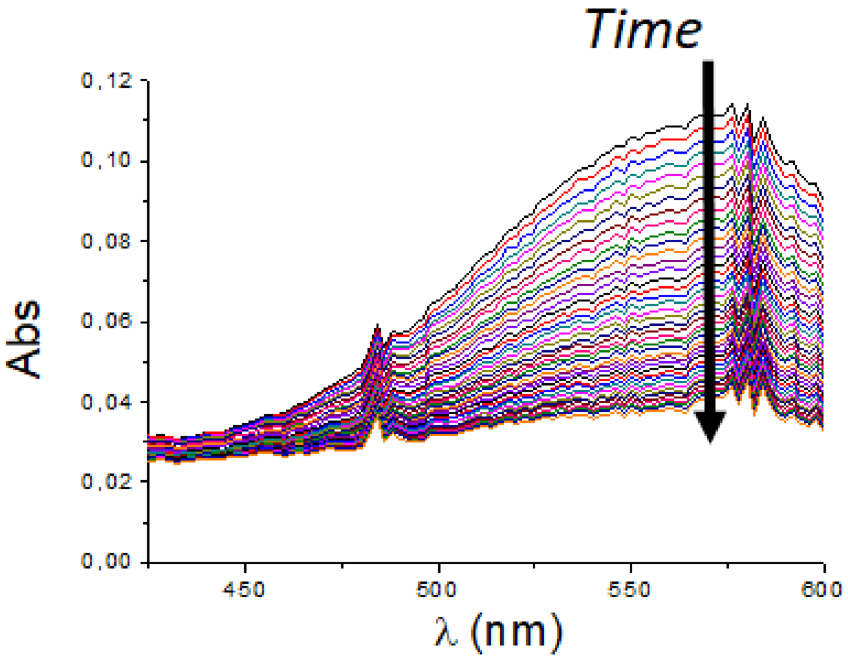

Figure S8-2: Photoisomerization of DAE induced by UCNP emission (zoom-in of the region of interest where only **1-c** absorbs).

In such conditions it is not possible to define simply the absorbed flux density [4]. Thus, no quantum yield could be determined. Nevertheless, the activity of the nanolamp could be determined: in this geometry, one single 35 nm-UCNP induces 20 dyes switching per second (ratio of the measured disappearance slope by the number of UCNP within the 976nm-irradiated volume).

9- Control experiments

a- Impact of the spectrophotometer lamp

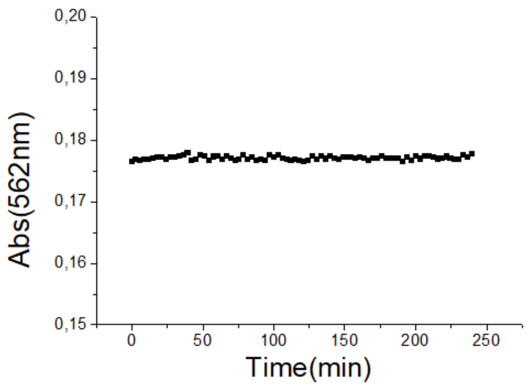

Figure S8-a : Time evolution of the absorbance of a mixture **1-c** and 35 nm-UCNP at 562 nm left in the spectrophotometer (no external light sources) ([**1-c**] = 1.6 10^{-5} mol L^{-1}, [UCNP] = 11g L^{-1})

b-Impact of the laser

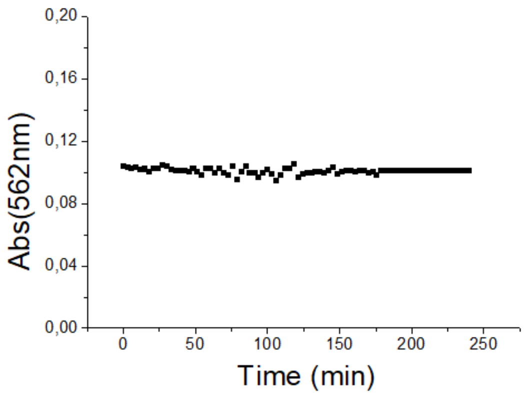

Figure S9-b: Time evolution of the absorbance of **1-c** at 562 nm under 980nm-laser irradiation ([**1-c**] = 9 10^{-6} mol L-1: P laser = 29 W cm^{-2})

Neither the spectrophotometer nor the 980-laser induce significant dye transformation.

10- Summary table

Step	parameter	symbol	unit	value
Photolysis of **1-c**	DAE **1-c** concentration	[**1-c**]	mol L^{-1}	2.12 × 10^{-4}
	**1-c** molar extinction coefficient at 650nm (monitoring)	ε_{650}	L mol^{-1} cm^{-1}	2154
	**1-c** molar extinction coefficient at 540nm	ε_{540}	L mol^{-1} cm^{-1}	9784
	Volume of the DAE solution	V	L	1.96 × 10^{-3}
	DAE opening quantum yield at 540nm	Φ_{CO}	-	0.02
	DAE Absorbance at 540 nm (irradiation)	Abs$_{540}$	-	2.07
	Optical length at 540 nm	L	cm	0.48
	Initial slope DAE photobleaching	-dAbs$_{650}$/dt	Abs s^{-1}	1.46 × 10^{-5}
	DAE bleaching rate	-d[**1-c**]/dt	mol L^{-1} s^{-1}	6.78 × 10^{-9}
	**DAE consumption**		**molecule s^{-1}**	8.00 × 10^{12}
Incoming NIR energy	Upconversion photon flux at 540nm	J_0	photon s^{-1}	4.00 × 10^{14}
	Laser power at 976 nm (NIR)	P	W	4.7
	Laser power density at 976 nm (NIR)		W cm^{-2}	49
	Incident NIR photon flux	J_0^{NIR}	photon s^{-1}	2.31 × 10^{19}
	Absorbance of UCNP solution at 976 nm	Abs$_{976}$	-	0.0014
	Absorbed NIR photon flux	J_a^{NIR}	photon s^{-1}	7.45 × 10^{16}
	**Up-conversion QY**	$\mathbf{\Phi_{UC}}$	**-**	**0.54%**
Single NP power	Laser beam section		cm^2	9.6 × 10^{-2}
	Laser beam volume inside the cell (“lamp”)	v	L	9.6 × 10^{-5}
	Yb molar extinction coefficient at 976 nm	ε_{Yb}	L mol^{-1} cm^{-1}	3.1
	number of Yb atoms inside the beam volume	n_{Yb}	atom	2.58 × 10^{16}
	Total number of RE per nanoparticle (from XRD)	N_{RE}	atom NP^{-1}	75490
	percentage of Yb	p_{Yb}	%	30
	percentage of Er	p_{Er}	%	2
	number of ytterbium per nanoparticle	N_{Yb}	atom NP^{-1}	22670
	number of erbium per nanoparticle	N_{Er}	atom NP^{-1}	1510
	number of NPs inside the laser beam	n_{Yb}	NP	1.14 × 10^2
	emitted photons per erbium atom	J_0/N_{Er}	photon s^{-1}	0.24
	**number of emitted photons per NP’s**	$\mathbf{J_0/n_{NP}}$	**photon s^{-1}**	**350**
	**Power per NP’s**	$\wp^{NP}$	**W**	**1.29 × 10^{16}**

11- References:

(1) Sumi, T.; Takagi, Y.; Yagi, A.; Morimoto, M.; Irie, M. *Chemical Communications* **2014**, *50* (30), 3928–3930. https://doi.org/10.1039/C4CC00396A.

(2) Li, Z.; Zhang, Y. *Nanotechnology* **2008**, *19* (34), 345606. https://doi.org/doi:10.1088/0957-4484/19/34/345606.

(3) Zhai, X.; Wang, Y.; Liu, X.; Liu, S.; Lei, P.; Yao, S.; Song, S.; Zhou, L.; Feng, J.; Zhang, H. *ChemPhotoChem* **2017**, *1* (8), 369–375.

(4) Aillet, T.; Loubiere, K.; Dechy-Cabaret, O.; Prat, L. *International Journal of Chemical Reactor Engineering* **2014**, *12* (1), 257–269. https://doi.org/10.1515/ijcre-2013-0121.
